# Epidemiological Trend Analysis of Bovine Tuberculosis and Its Public Health Impact in Ethiopia

**DOI:** 10.1155/jotm/2727632

**Published:** 2025-10-05

**Authors:** Dawit Gebremichael

**Affiliations:** Department of Animal Science, Aksum University, Shire, Ethiopia

**Keywords:** bovine tuberculosis, risk factor, trends, zoonotic impact

## Abstract

Bovine tuberculosis (bTB) has significant economic losses on the livestock productivity and poses serious public health risks worldwide. In Ethiopia, bTB is endemic and distributed across all parts of the country. Therefore, the systematic review aims to provide comprehensive investigations of the trends, risk factors, and zoonotic impacts of bTB in Ethiopia. Only English language publications from 2009 to 2022 were included. Databases searched included PubMed, PubMed Central, Medline, Scopus, Web of Science, Google Scholar, and ResearchGate. From a total of 129 articles retrieved, only 44 studies fulfilled the eligibility criteria. The overall pooled prevalence of bTB over 15 years was 11.04%. Female cattle had a significantly higher prevalence (19.85%) compared to male cattle (4.07%) (*p*=0.002). Emaciated animals were more affected than those in good body condition (*p*=0.04). Prevalence differed significantly among intensive (22.60%), semi-intensive (17.08%), and extensive (13.70%) production systems (*p*=0.01). The prevalence of bTB showed statistical significance (*p*=0.001) in three different breeds. Exotic breeds had the highest prevalence (28.46%), followed by crossed (12.61%) and local breeds (9.56%). Large herds showed a higher prevalence (42.69%) compared to medium (12.13%) and small herds (11.26%) (*p*=0.001). Poor management systems had a higher positivity rate (31.27%) compared to medium (15.23%) and good (9.89%) management systems (*p*=0.01). Studies with sample sizes less than 400 reported higher prevalence (20.70%) compared to those with 400–800 samples (11.70%) and more than 800 samples (5.83%) (*p*=0.001). Significant variations were observed among different diagnosis techniques (*p*=0.004). Only 40.82% and 35.51% of cattle owners had knowledge of bTB and awareness of its zoonotic impacts, respectively. Overall, the trends of bTB remain a significant impact in Ethiopia, affecting both livestock productivity and public health. Understanding these trends is essential for the development of evidence-based control strategies. The integration of epidemiological modeling with public health impact assessments can further strengthen policy formulation and guide resource prioritization more effectively. Therefore, effective control and prevention measures, along with continuous public education, are essential to mitigate economic losses and reduce the zoonotic impacts of bTB in the country. Using improved diagnostic techniques should be recommended to estimate the actual prevalence of the disease.

## 1. Introduction

Bovine tuberculosis (bTB) is a chronic and contagious disease of cattle and other domestic and wild animals [1–5]. bTB is caused by *Mycobacterium bovis* (*M. bovis*) [6], and it is distributed all over the world [5, 7, 8], with a significant economic impact on the livestock production sector in developing countries [9–11]. The economic impact of bTB on livestock production, particularly in developing countries, is substantial, as it can lead to reduced productivity, trade restrictions, and increased costs associated with disease control measures [12]. The estimated losses of around 3 million USD annually underscore the severity of the issue and the need for effective management strategies [13].

Moreover, the zoonotic potential of bTB underscores its significance for public health. The World Health Organization (WHO) reported that 147,000 cases and 12,500 deaths happened worldwide from zoonotic bTB [7, 14, 15]. However, the lack of comprehensive surveillance data, particularly from developing regions, likely underestimates the true burden of bTB on human health. Improved surveillance efforts are essential for accurately assessing the disease's impact and implementing targeted interventions to mitigate its spread [16].

Human immunodeficiency virus (HIV) is a major risk factor for the development of active tuberculosis (TB), particularly in developing countries. HIV selectively depletes CD4+ T lymphocytes, which play a central role in coordinating immune responses against intracellular pathogens such as *M. bovis*. This depletion impairs the production of Th1-type cytokines, which are critical for activating macrophages, promoting phagolysosomal fusion, and maintaining the integrity of granulomas—key defenses against mycobacterial infection. In HIV-infected individuals, macrophages exhibit reduced phagolysosomal fusion and impaired antigen presentation, significantly weakening the initial immune response to *M. bovis*. As a result, latent bTB infections reactivate 5 to 15 times more frequently in HIV-positive individuals due to compromised immune surveillance. Malnutrition independently contributes to CD4+ T cell depletion and further exacerbates HIV-induced immunosuppression. In sub-Saharan Africa, where HIV and malnutrition frequently coexist, individuals may face a greater than 30% risk of developing active bTB. Moreover, coinfection with HIV and bTB is associated with mortality rates exceeding 50% in the absence of antiretroviral therapy (ART) [17–19].

In African countries, the proportion of TB cases coinfected with HIV was 39%, which accounted for 79% of TB cases among people living with HIV worldwide [19]. Ethiopia is one of the 22 countries with a high burden of TB, which together account for 80% of all global TB burden. Ethiopia is the second most infected country with HIV in Africa, next to South Africa [20–23]. Combating HIV/AIDS is one of the millennium development goals. However, in order to realize this goal, bTB eradication is considered as the main challenge for the future [17]. It has been recorded as the most recurrent cause of zoonotic bTB in humans [11, 24, 25]. In addition, with the emergence of new dairy hubs across the country, a valid worry is the spread of bTB to other regions, which might increase the risk for zoonotic TB in the country. The transmission of bTB from cattle to humans occurs through consumption of infected animal products or close contact with infected animals, posing a risk to public health [15]. Moreover, Ethiopia's growing dairy industry poses extra concerns regarding the increasing trends of bTB. Consuming habits of raw milk and meat (Qurt) are common practices in the pastoralists and central highlands of the country, respectively. These habits can lead to the likelihood of human exposure to *M. bovis*, further complicating efforts to control the disease. Different research findings indicated that *M. bovis* causes 10%–15% of human cases of TB in country [26].

bTB is an endemic disease of cattle in Ethiopia and is distributed in almost all parts of the country. The challenges of bTB include inadequate surveillance systems, limited resources for control measures, and socioeconomic factors affecting disease spread [11]. Many studies have stated that there are many potential associated risk factors that are conducive to the spreading and persistence of bTB in the country [27, 28]. Some of the risk factors include lack of knowledge of the transmission and distribution methods of the pathogen, poor public awareness of zoonotic impact of the disease, consumption practices, demography characteristics, socioeconomic status, and using the same house for humans and animals [24, 29]. The prevalence of bTB in Ethiopia was found to be high ranging from 3.5% to 70% depending on the geographical areas, the breeds, and the husbandry practices [1, 30–32]. The disease causes significant economic losses in the country. Indeed, bovine tuberculosis causes substantial economic losses through reduced livestock productivity, international trade restrictions, expenses related to control and surveillance measures, and its impact on human health [12]. The bTB-diseased animal loses 10%–25% of their productive efficiency; direct losses due to the infection become evident by a decrease in 10%–18% milk and a 15% reduction in meat production, overall loss of 131,709,530 USD in the country [11, 26].

Despite the significant impact of bTB, there is a lack of comprehensive systematic reviews on the topic in Ethiopia. Existing reviews often lack detailed descriptions of risk factors, trends, and public health impacts. Moreover, discrepancies in prevalence estimates among studies using different methodologies, such as comparative intradermal tuberculin test (CIDT), postmortem inspection (PMI), and culture, highlight the need for standardized approaches and better methodological comparisons.

Therefore, the aim of conducting a systematic review on bTB in Ethiopia is to provide a comprehensive investigation of the disease's status, pooled prevalence, associated risk factors, and public health impacts. Such a review would help to fill the knowledge gaps, inform policy decisions, and guide future research and control efforts. Additionally, comparing and describing the types of methodologies used in previous studies and their respective prevalence estimates and associated risk factors would enhance our understanding of the disease dynamics and inform best practices for surveillance and control. Understanding these trends is crucial for developing effective control strategies and mitigating the public health impact of bTB in Ethiopia.

## 2. Methodology

### 2.1. Search Strategy

The databases used for this review included PubMed, PubMed Central, Web of Science, Medline, Scopus, Google Scholar, ResearchGate, and HINARI. To obtain relevant studies, the following keywords were used during the database search: bTB, ^∗^prevalence of bovine tuberculosis, risk determinants of bTB, public health impacts, M. bovis, and Ethiopia. Only publications in English language and published between 2009 and 2022 were considered. However the study periods from 2008 to 2021 were included in the review.

### 2.2. Inclusion and Exclusion Criteria

Titles and abstracts were reviewed to retrieve studies on the bTB. Articles found relevant by title and abstract were subjected to full-text review for eligibility. Only research articles published on bTB in English language, with full texts and clear methodology, were included in the review, whereas articles in other languages, case reports, abstracts, proceedings, and unpublished articles were excluded.

### 2.3. Data Extraction

The data and information were extracted from all the eligible studies including authors, geographical location, diagnosis techniques, region, year of study, year of publication, sample size, prevalence of bTB, and associated risk factors with bTB such as age, sex, body condition, production system, breeds, herd size, and management system. Data and information extracted also included public health impacts of bTB such as having awareness and knowledge of zoonotic impact about bTB and consuming habits of raw milk or meat.

### 2.4. Statistical Analysis

After coding and editing, the data were imported to SPSS Version 20. A pooled prevalence was estimated. Pearson's chi-square tests and odds ratios were calculated on associated risk factors such as geographical location, diagnosis techniques, region, year of study, year of publication, sample size, prevalence of bTB, age, sex, body condition, production system, breeds, herd size, and management system.

## 3. Results

A total of 129 articles were retrieved from PubMed, Medline, Scopus, Web of Science, Google Scholar, ResearchGate, etc. Of which, 86 were nonduplicate and subjected to further evaluation and 44 of them were excluded based on the title and abstract evaluation. Finally, 44 studies fulfilled the eligibility criteria and were involved in the review.

Study characteristics were focused on geographical location, diagnosis techniques, year of study, year of publication, sample size, prevalence of bTB, and potential associated risk factors of bTB such as age, sex, body condition, production system, breeds, herd size, and management system. In addition, the review also was assessed on awareness and knowledge of zoonotic impact about bTB and consuming habits of raw milk or meat. The overall pooled prevalence of bTB over these 15 years was found to be 11.04%, with individual study estimated ranging from 0.1% to 54.6% (Table 1).

### 3.1. Potential Associated Risk Factors

Data of sex of animals were analyzed to identify whether sex is a potential associated risk factor. A statistically significant difference was observed between bTB pooled prevalence and sex categories. Female cattle were significantly more affected by bTB than male cattle (*p*=0.002). The overall pooled prevalence of female and male was found to be 19.85% and 4.07%, respectively. Concerning breeds types, the pooled prevalence of bTB showed statistical significance (*p*=0.001) in three different breeds. Exotic breeds had the highest prevalence (28.46%), followed by crossed (12.61%) and local breeds (9.56%). Body condition score (BCS) was categorized into three scales: poor, medium, and good, a modification from the three scales described by Kellogg to better reflect the assessment in field conditions. In the present study, statistically significant (*p*=0.04) difference in the occurrence of bTB was revealed between different classes of BCS. Emaciated animals were more affected by bTB than medium and good body conditions (Table 2).

The result indicated that production systems were risk factors of bTB prevalence. Pooled prevalence of bTB was observed to have statistically significant differences among different production systems (*p*=0.01). Overall pooled prevalence of bTB differed significantly among intensive (22.60%), semi-intensive (17.08%), and extensive (13.70%) production systems. The result identified herd size as one of the major risk factors of bTB. Large herds (> 50 cattle) showed a higher prevalence (42.69%) compared to medium (10–50 cattle) (12.13%) and small herds (< 10 cattle) (11.26%) (*p*=0.001). The pooled prevalence of bTB was compared among different management systems. Management systems were significantly associated with bTB reactivity (*p*=0.01). Poor management systems had a higher positivity rate (31.27%) compared to medium (15.23%) and good (9.89%) management systems (*p*=0.01). The age of the study animals was also determined according to Daraje et al. [8]. The pooled prevalence of bTB was not significantly different among age categories (*p*=0.6) (Table 2).

### 3.2. Trends of bTB

**Figure 1:**
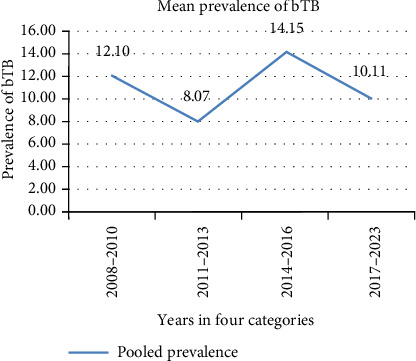
Trends of pooled prevalence of bTB over 15 years.

The 15-year trend analysis revealed a slightly different pattern. The highest prevalence of bTB was observed from 2014–2016, with a rate of 14.15%, and that from 2011–2013 was 9.56%, the lowest in the 15-year span (Figure 1).

### 3.3. Geographical Location, Diagnostic Techniques, and Sample Size

The result tried to compare the prevalence of bTB on different geographical locations across the country. Central Ethiopia had the highest prevalence (17.99%), followed by Eastern Ethiopia (16.32%), with the lowest in Northwestern Ethiopia (3.74%) (*p* = 0.02). Association was also measured among the diagnosis techniques; there was significant variation in prevalence among different diagnostic techniques: comparative intradermal test (15.10%), PMI (6.47%), and cultures (2.52%) (*p* = 0.004). The sample size was calculated to identify statistically significant associations. Studies with sample sizes less than 400 reported higher prevalence (20.70%) compared to those with 400–800 sample sizes (11.70%) and more than 800 sample sizes (5.83%) (*p* = 0.001) (Table 3).

### 3.4. Knowledge and Public Awareness

**Figure 2:**
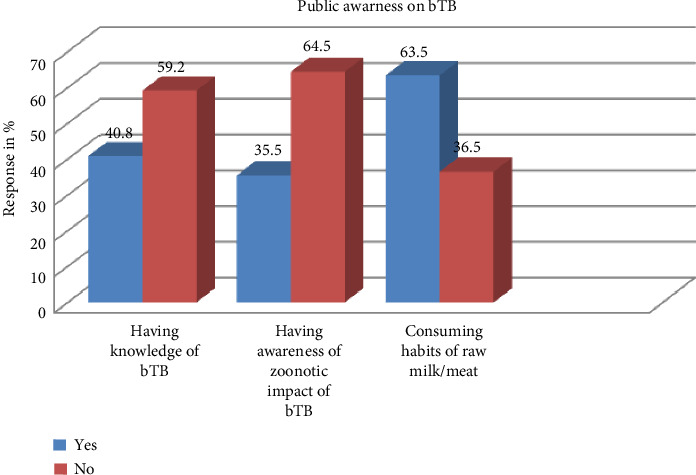
Trends of public awareness on bTB.

Only 40.8% and 35.5% of the cattle owners had overall knowledge of bTB and awareness of zoonotic impact of bTB, respectively. On the other hand, the majority of (63.5%) cattle owners consumed raw milk/meat (Figure 2). Consuming habits of raw milk/meat ranged from 15% to 89.8% (Table 4).

## 4. Discussion

A 15-year trend analysis was conducted to determine the trends, prevalence, associated risk factors, and public health impacts of bTB in Ethiopia. The study recorded a high burden of bTB in both cattle and humans within the country [6, 9, 20, 62]. The overall pooled prevalence of bTB over these 15 years was found to be 11.04%, with individual study estimated ranging from 0.1% [1] to 54.6% [9]. This wide range in prevalence can be attributed to differences in diagnostic techniques, study methodologies, sample sizes, and geographical locations used by various researchers and authors.

The results indicated that sex of the animal, breed types, body conditions, production systems, herd size, and management systems were the potential risk factors of bTB. Female cattle were significantly more affected by bTB than male cattle (*p*=0.002). The pooled prevalence in female and male cattle was found to be 19.85% and 4.07%, respectively. Most researchers conducted their studies in dairy farms and abattoirs, particularly in Central Ethiopia. Almost all dairy farms in this region consist primarily of female cattle managed in intensive farming systems, while most animals slaughtered in abattoirs are male cattle managed under extensive farming systems. Additionally, the majority of dairy cows in Central Ethiopia are exotic breeds, which are more susceptible to bTB compared to local breeds [30]. Intensive farming systems, which are characterized by high animal density and stress levels, make dairy cows more vulnerable to bTB than those managed under extensive systems. The stress and overcrowding associated with intensive farming exacerbate the susceptibility of dairy cattle to bTB [57]. The prevalence of bTB in dairy cattle in Central Ethiopia ranges between 30% and 40% on average, with some farms experiencing prevalence rates as high as 70% [9, 63].

The pooled prevalence of bTB did not show significant differences among age categories. However, some reports indicated a significant association between age and bTB prevalence in the country. Older animals were found to be more reactive to bTB than younger animals [31, 45, 63]. Similarly, Kapalamula et al. [5] also stated from Malawi that older animals were more likely to present with bTB-like lesions at slaughter than younger animals. This increased risk in older animals is attributed to prolonged exposure to the pathogen in the environment and the physiological decline of their immune systems over time [24].

The mean prevalence of bTB showed a statistically significant difference (*p*=0.001) among three different breeds. The evidence suggests a significant association between cattle breeds and their susceptibility or resistance to bTB. Exotic breeds, such as Holstein-Friesians (HFs), were found to be the most susceptible to bTB compared to crossbred and local cattle breeds (*p*=0.001). Crossbred cattle were also more susceptible than local breeds. Studies conducted in Ethiopia have shown that both the prevalence and severity of TB lesions are higher in HF cattle compared to zebu cattle. Genetic differences between cattle breeds play a significant role in determining their susceptibility or resistance to bTB [5, 26, 64, 65]. Differences in bTB prevalence among cattle breeds may be attributed to variations in innate immunity, particularly those related to the major histocompatibility complex (MHC) genes. Zebu cattle (*Bos indicus*), which evolved in tropical regions under high pathogen pressure—including exposure to mycobacteria—exhibit greater natural resistance to bTB. This resistance may also be linked to their lower basal cortisol levels and reduced physiological stress reactivity.

In contrast, HF cattle are highly susceptible to bTB. Chronic stressors such as heat, intensive handling, and confinement, which are common in intensive dairy systems, suppress immune responses and increase susceptibility. Moreover, HF breeds have been intensively selected for high milk yield and rapid growth, resulting in resource allocation trade-offs—diverting metabolic energy away from immune function and toward production. This imbalance heightens vulnerability to infections, including bTB [9].

During peak lactation, HF cows experience severe metabolic stress, marked by elevated cortisol levels and suppressed immune responses—further compromising the ability to control *M. bovis* infection. These physiological trade-offs, compounded by management-related stressors, explain the increased risk of bTB in HF cattle compared to more resilient indigenous breeds like zebu [12, 42, 65]. Similarly, Kapalamula et al. [5] reported that improved breeds seem to be more susceptible to bTB than local breeds. Overall, the breed of cattle has been identified as a significant risk factor for bTB, with imported breeds being particularly vulnerable. Studies have highlighted the importance of breed-specific factors in determining susceptibility to bTB.

The association between body condition and bTB infection is significant, with emaciated animals being more affected than those in good body condition. The pooled prevalence analysis revealed statistically significant variations in bTB prevalence among different body conditions, with emaciated animals showing higher rates of infection. This suggests a strong association between poor body condition and bTB infection. Poor BCS is identified as a risk factor for bTB infection. Hussein et al. [60] reported that animals with poor BCS had a risk 11.4 times higher of bTB than those with good BCS. Animals with a low BCS are more susceptible to bTB infection, possibly due to compromised immune function or increased susceptibility to disease. Malnutrition (especially protein and vitamin A deficiency) damages the integrity of the respiratory and gastrointestinal tract linings. This makes it easier for *M. bovis* to invade tissues upon exposure [17]. Malnourished animals often have lower levels of circulating immune cells and antibodies. The immune response is slower and weaker when challenged by *M. bovis*. The crucial cell-mediated immune (CMI) response (T-cells activating macrophages to kill the bacteria inside them) is particularly impaired by deficiencies in protein, energy, vitamin A, vitamin D, and zinc. This is the primary defense needed to control bTB infection [56]. Malnutrition prevents this activation, allowing the bacteria to survive and multiply unchecked within macrophages [12]. Additionally, malnourished animals with suppressed immunity may show false-negative results because their immune system is too weak to mount a detectable reaction, even if infected. This allows infected, malnourished animals to remain undetected in the herd, spreading the disease.

The findings regarding the prevalence of bTB in different production systems such as intensive, semi-intensive, and extensive systems provided valuable insights into the relationship between production practices and disease transmission. The overall mean prevalence of bTB was highest in intensive production systems (22.60%), followed by semi-intensive (17.08%) and extensive systems (13.70%). This indicated a significant difference in bTB prevalence among different production systems, with intensively managed cattle showing the highest prevalence rates. Intensive production systems, characterized by high stocking densities, stressed animals, and overcrowding, are associated with higher bTB prevalence [52, 66]. Close contact among animals facilitates disease transmission, particularly through respiratory routes. Studies have consistently found that the highest incidence of bTB is generally found in areas where intensive dairy systems are practiced, where close contact among cattle is common [9, 17, 35, 46, 65]. Cattle kept under intensive farming systems had more reactors and more severe pathology [67] because of their closer confinement, longer life spans, and higher levels of productivity stress [61]. Overall, the findings highlight the importance of production practices in influencing the prevalence of bTB in cattle populations. Intensive production systems, characterized by close contact among animals and stress factors, are associated with higher bTB prevalence rates compared to extensive systems. Implementing measures to reduce overcrowding, improve biosecurity, and mitigate stress factors can help minimize the spread of bTB in intensively managed cattle populations.

The results identified herd size as one of the major risk factors for bTB. Large herd sizes harbored the disease significantly more than medium and small herd sizes (*p*=0.001). High stocking densities facilitate the increased transmission of infectious pathogens due to overcrowding and poor ventilation [6, 12, 27]. As herd size increases, the probability of acquiring of bTB increases for these animals are kept in an indoor system [9, 31, 35, 67, 68].

The findings highlight a significant association between management systems and the prevalence of bTB, with poor management conditions being linked to higher rates of bTB positivity. The review found a significant association between different management systems (poor, medium, and good) and bTB positivity. Animals kept under poor management conditions showed a higher prevalence of bTB compared to those under good husbandry systems. Farms with poor management conditions may facilitate the persistence of *M. bovis* infection, creating a suitable environment for easy multiplying and transmission [6]. According to Kemal et al. [24], poorly managed farms had a 3.7 times higher risk of bTB compared with well-managed farms. This indicates the importance of implementing effective hygiene and management practices to mitigate the spread of bTB in intensive farming settings. In Ethiopia, different authors observed that poor hygienic practices can result in the reduction of an animal's resistance to bTB [17]. Overall, the findings underscore the importance of addressing management practices, hygiene, and housing conditions to control the spread of bTB in cattle populations. Hygiene and sanitation practices, including manure disposal, drainage systems, and barn cleaning frequency, are crucial for preventing bTB transmission [9, 12, 17, 31, 35, 46, 65].

Bovine TB prevalence exhibited a slight decline over time. But still significant incidences were recorded in the countries. The highest bTB prevalence (14.15%) was found from 2014 to 2016. Geographical locations had association with bTB prevalence. The highest prevalence was observed in Central Ethiopia (17.99%), followed by Eastern Ethiopia (16.32%), with the lowest in Northwestern Ethiopia (3.74%). This may be due to the fact that Holstein cattle are progressively imported to improve milk production, especially in the central part of the country [17]. The area has a significant dairy cattle population and it serves the main source of improved dairy cattle to the rest of the country [9]. However, comparing geographical locations using different diagnostic methods and sample sizes is not suitable for drawing conclusive results. Risk factors that are imperative for the occurrence of bTB, particularly those related to human activities and intra-species transmission of *M. bovis*, seem to be more significant, especially in areas where intensification practices are employed [1, 9, 35]. The review results also assessed significant associations of diagnosis techniques, sample size, and geographical locations with bTB positivity. The highest prevalence (15.10%) was observed with the CIDT in the field, followed by 6.47% in PMI at abattoirs and 2.52% in cultures in laboratories. There are various reasons why significant differences are observed in different diagnostic techniques for bTB. The CIDT is widely recognized as the standard method for live-animal screening for bTB due to its high specificity, provided that it is correctly administered and interpreted [65]. However, CIDT is limited by low sensitivity and can produce false-positive results, particularly in animals with a history of BCG vaccination, exposure to nontuberculous mycobacteria, or in cases of misinterpretation [17].

Moreover, certain animal conditions compromise CIDT reliability. Severely malnourished, stressed, or coinfected cattle may fail to mount a detectable CMI response. In early or latent infections, insufficient bacterial load or immune priming (typically within 4–6 weeks postexposure) limits test reactivity. In advanced disease, immune exhaustion may blunt responsiveness. Additionally, endogenous or exogenous corticosteroids can suppress immune reactivity, further contributing to false-negative results. These factors can render infected animals unresponsive to CIDT, even in advanced disease stages, leading to underestimation of true prevalence [13, 17, 34, 45, 51, 67].

PMI remains a routine method in abattoirs for detecting bTB-associated lesions. Despite its widespread use, PMI has low sensitivity. Studies indicate that 30%–50% of cattle with confirmed *M. bovis* infection (as verified by culture) show no visible lesions at slaughter. This is common in early infections (< 60 days postexposure), latent or paucibacillary forms, and in genetically resistant breeds such as zebu [13, 34, 43]. Even when lesions are present, a significant number are overlooked during routine inspection, especially extrapulmonary or small lesions. The overall sensitivity of PMI has been reported at just 28.2%, indicating that it substantially underestimates the prevalence of bTB. For more accurate detection, systematic tissue sampling for histopathology or culture is required [63].

Culture methods are considered the gold standard for confirming *M. bovis* infection due to their high specificity and ability to confirm viable organisms. However, culture is slow, labor-intensive, and technically demanding. False negatives can occur due to sample contamination, or if *M. bovis* enters a metabolically inactive (latent) state, making it undetectable in standard media.

Given the limitations of each diagnostic approach, no single test can detect all infected animals due to the biological complexity of TB, including latency and immunosuppression. Therefore, a multimodal diagnostic strategy is essential. Combining CIDT, PMI, acid-fast staining, and culture can enhance the overall diagnostic accuracy for bTB. Furthermore, the development and integration of molecular techniques, such as PCR-based assays, offer promise for detecting latent infections and improving surveillance accuracy [17, 42].

The results showed that the prevalence of bTB varied statistically according to sample size categories. Researchers using small sample sizes reported higher prevalence of bTB positives compared to those using large sample sizes. The use of sample size calculation directly influences research findings. Very small samples undermine the internal and external validity of a study. The sample size dictates the amount of information we have and, therefore, in part, determines our precision or the level of confidence we have in our sample estimates. An estimate always has an associated level of uncertainty, which depends upon the underlying variability of the data as well as the sample size. As sample size increases, confidence in our estimate increases, uncertainty decreases, and we achieve greater precision. Increasing our sample size can also give us greater power to detect differences. Larger sample sizes provide more reliable results with greater precision and increase the probability of finding statistically significant evidence of a difference between groups, assuming a difference exists in the population. This allows researchers to draw more meaningful conclusions.

Concerning knowledge and zoonotic impacts of bTB, the review results revealed that cattle owners have a low level of knowledge about bTB and are unaware of its zoonotic impacts. Even among those who are aware of bTB, many do not understand how transmission occurs from animals to humans [23]. Additionally, a high proportion of cattle owners reported consuming raw milk and meat. However, various studies have identified the consumption of raw milk and meat as potential transmission routes for bTB to humans [23, 67]. In Central Ethiopia, the consumption of raw meat (known as Qurt) is a common practice in both rural and urban areas, including the capital city. Similarly, drinking raw milk is highly prevalent in pastoral areas and other parts of the country [6, 24]. According to Hussein et al. [60], the habit of consuming raw milk was common in pastoral area; almost all (89.2%) of the pastoralists had no awareness of the likelihood of bTB transmission through milk. *M. bovis* is primarily transmitted to humans through the consumption of unpasteurized milk or undercooked meat and less commonly via inhalation of aerosols from infected animals. The mucosal surfaces of the oropharynx and respiratory tract act as key entry points for the pathogen. The viability of *M. bovis* in raw milk and its ability to survive traditional food processing methods pose a significant public health concern. The bacterium can persist in raw milk for extended periods and is capable of surviving traditional fermentation practices, including the preparation of yogurt, cheese, and butter. In Ethiopia, the traditional methods of preparing dairy products—particularly cheese and butter—may increase the risk of zoonotic bTB transmission, as *M. bovis* can remain viable throughout these processes [20]. Moreover, studies have reported that a large proportion of cattle owners lack awareness about bTB transmission routes and often share living spaces with their livestock, further facilitating the spread of the disease [23]. Under such conditions, *M. bovis* has been shown to survive for several weeks to months, increasing the likelihood of human exposure.

The low level of knowledge, unawareness of the zoonotic consequences of bTB, food consumption behaviors, and poor hygienic practices are significant risk factors for bTB transmission to public health [24]. Community-based public health education remains a crucial tool in raising awareness among cattle owners [23]. Currently, global efforts are underway to address zoonotic bTB as part of the goal to end the global TB epidemic by 2030. However, in Ethiopia, there is a lack of policies and implementation activities aligned with these global endeavors for the control of bTB [7]. Incorporating awareness of these mechanisms is crucial for public health messaging.

## 5. Conclusions

Based on the results of 15-year trend analysis, bTB is highly prevalent in Ethiopia, with significant risk factors including sex of the animal, body condition of the animal, production systems, types of breeds, herd size, and management systems. Additionally, cattle owners exhibit a low level of knowledge about bTB and awareness of its zoonotic impacts. Consumption habits of raw meat and milk are widespread throughout the country. Understanding these trends is crucial for developing evidence-based control strategies. Moving forward, the integration of epidemiological modeling and advanced mathematical tools is essential for improving the control and prevention of bTB. Compartmental models, such as the susceptible-infectious-recovered (SIR) framework, along with optimal control models, provide valuable insights by simulating disease dynamics in cattle herds under varying management scenarios. These models support the design and evaluation of intervention strategies—including testing regimes, culling, vaccination, movement restrictions, and housing improvements—prior to implementation. This approach enables policymakers and veterinary health authorities to forecast potential outcomes, optimize resource allocation, and identify the most effective and cost-efficient control strategies. Furthermore, incorporating disability-adjusted life years (DALYs) to quantify the public health burden of zoonotic bTB facilitates evidence-based prioritization within broader health agendas, aligning with the One Health approach. For countries like Ethiopia, particularly in high-risk regions, adopting these predictive and evaluative tools in bTB research and surveillance programs could significantly enhance disease monitoring, control efficiency, and cross-sectoral coordination. Integrated multidisciplinary teams should be established to conduct surveillance, monitoring, and evaluation efficiently. Continuous training and public education programs are critical to raise awareness about the zoonotic impacts of bTB, sources of infection, and means of transmission. Special emphasis should also be placed on educating cattle owners about effective dairy farm management systems to mitigate the spread of bTB. These efforts are essential for improving public health outcomes and reducing the prevalence of bTB in both cattle and human populations in Ethiopia. Finally, using improved diagnostic techniques should be recommended to estimate the actual prevalence of the disease.

## Figures and Tables

**Table 1:** Individual prevalence of bTB over 15 years.

Authors	Study year	Study area/district	Methodology	Sample size	Prevalence
[1]	2008	Gonder	Culture and ZN	14,314	0.1
[1]	2008	Woldiya	Culture and ZN	4338	0.4
[1]	2008	Jinka	Culture and ZN	3471	1.6
[1]	2008	Addis Ababa	Culture and ZN	2800	0.6
[1]	2008	Butajira	Culture and ZN	4606	0.6
[1]	2008	Gimbi	Culture and ZN	3250	1.1
[33]	2008	Arisi Negele	CIDT	425	6.4
[31]	2008	Hawasa	CIDT	413	11.6
[34]	2009	Hamer	CIDT	499	4.2
[35]	2010	Addis Ababa	CIDT	1132	34.1
[36]	2010	Arisi Negele	CIDT	625	12.2
[37]	2011	Addis Ababa	PMI	720	5.8
[9]	2011	Addis Ababa	CIDT	758	23.1
[9]	2011	Debrezeit	CIDT	649	22.8
[9]	2011	Sebeta	CIDT	449	54.6
[9]	2011	Sululta	CIDT	398	47.7
[9]	2011	Holeta	CIDT	361	9.4
[9]	2011	Sendafa	CIDT	341	42.5
[38]	2011	Mekelle	CIDT		13.9
[39]	2009	Liben zone	CIDT	411	2.4
[40]	2008	Jimma	PMI	1102	5.4
[41]	2008	Jimma	CIDT	384	21.4
[42]	2010	Selalle	CIDT	5424	2.82
[20]	2010	Fiche	CIDT	2033	6.5
[43]	2010	Bako	PMI	487	9.3
[21]	2010	Woldiya	PMI	1029	6.1
[44]	2013	Horro Guduru	CIDT	500	1.8
[45]	2011	Afar	CIDT	1087	11
[46]	2011	Humera	CIDT	484	6.6
[47]	2011	Mekelle	PMI	768	6.4
[48]	2012	Mekelle	CIDT	423	7.3
[49]	2013	Sululta	CIDT	858	11.4
[49]	2013	Sululta	PMI	1107	3.5
[49]	2013	Sululta	Culture and ZN	39	7.7
[50]	2013	Dilla	PMI	768	2.6
[51]	2011	Adama	PMI	486	6.8
[52]	2013	Nekemte	PMI	1183	5.7
[53]	2011	Mekelle	CIDT	480	11.3
[54]	2013	Gonder	CIDT	364	8.7
[54]	2013	Woldiya	CIDT	117	15.3
[55]	2015	Gambella town	PMI	500	13.2
[56]	2013	Debrezeit	CIDT	558	17.0
[27]	2015	Awash Park	CIDT	2550	5.5
[57]	2015	Adama	CIDT	501	4.4
[22]	2016	Borana zone	CIDT	554	3.8
[58]	2015	Bahir Dar	CIDT	2846	2.78
[15]	2018	Gonder	CIDT	315	1.59
[24]	2016	Harar	CIDT	224	11.2
[24]	2016	Dire Dawa	CIDT	58	50
[24]	2016	Jigjiga	CIDT	33	30.3
[12]	2017	Mekelle	CIDT	818	12.0
[12]	2017	Gonder	CIDT	976	1.4
[12]	2017	Hawassa	CIDT	960	3.0
[10]	2010	Woldiya	PMI	1029	6.1
[59]	2009	Gimbi	PMI	400	7.8
[59]	2009	Gimbi	Culture and ZN	31	12.9
[3]	2018	Gonder	PMI	497	9.1
[3]	2018	Gonder	Culture and ZN	45	4.4
[6]	2019	Addis Ababa	CIDT	654	39.3
[60]	2021	Fafan	CIDT	249	11.2
[61]	2022	Hawasa	CIDT	202	20.3

**Table 2:** Potential associated risk factors of bTB.

Variables		Mean prevalence	SE for mean	95% CI for mean		*p* value
				Lower bound	Upper bound	
Sex of the animal	Male	4.07	1.11	1.36	6.79	0.002
	Female	19.85	12.84	−11.57	51.26	

Age of the animal in years	Less than 5	8.61	2.54	2.94	14.28	0.6
	Between 5 and 10	12.84	2.80	6.61	19.07	
	Greater than 10	8.22	1.45	4.98	11.45	

Body condition score	Emaciation	15.25	3.23	8.04	22.45	0.04
	Medium	10.38	1.88	6.19	14.58	
	Good body condition	10.36	2.52	4.75	15.98	

Production system	Intensive	22.60	0.80	20.05	25.15	0.01
	Semi-intensive	17.08	2.93	7.77	26.38	
	Extensive	13.70	0.00	13.70	13.70	

Breeds	Local	9.56	3.04	2.13	17.00	0.000
	Crossed	12.61	4.38	1.90	23.32	
	Exotic	28.46	8.45	5.01	51.91	

Herd size	Less than 10	11.26	3.59	2.48	20.04	0.001
	Between 10 and 50	12.13	2.42	6.21	18.05	
	Greater than 50	42.69	12.13	13.02	72.36	

Management system	Good	9.89	3.29	−0.57	20.36	0.01
	Medium	15.23	4.85	−0.22	30.67	
	Poor	31.27	5.67	13.22	49.31	

**Table 3:** Prevalence of bTB on geographical location, diagnostic techniques, and sample size.

Variables		Pooled prevalence	SE for pooled prevalence	95% CI for pooled prevalence		*p* value
				Lower bound	Upper bound	
Geographical location	Northern	8.31	1.35	5.27	11.35	0.02
	Eastern	16.32	6.64	0.08	32.56	
	Southern	6.72	1.56	3.31	10.12	
	Western	7.15	1.83	2.67	11.62	
	Central	17.99	3.98	9.64	26.35	
	Northwestern	3.74	1.21	0.89	6.59	

Diagnosis techniques	CIDT	15.10	2.40	10.23	19.97	0.004
	PMI	6.47	0.80	4.78	8.15	
	Culture	2.52	0.95	0.34	4.70	

Sample size	Less than 400	20.70	5.05	9.58	31.82	0.001
	400–800	11.70	2.44	6.66	16.75	
	Greater than 800	5.83	1.35	3.05	8.62	

**Table 4:** Individual reports of public awareness on bTB.

Authors	Having knowledge of bTB in %	Having awareness of zoonotic impact of bTB in %	Consuming habits of raw meat/milk in %	Having awareness close contact with cattle can facilitate respiratory/mucosal transmission	Awareness about living in close contact with cattle as sources of infection
[33]	6.9	34.7	58.2		
[9]	71.2	—	—		
[20]	61.6	27.4		27.40	33.60
[49]		10.3	79.3		
[53]	22.8	30.8	15		
[55]	22	15	45	3.3	15
[56]	33.3	33.3	83.3	30	
[22]	30	11.5	79.2		
[58]	34.3	27.14	61.7		
[14]	95.3	93	—		
[15]	32	32	69.4		
[24]	32.55	23.25	53.48	11.62	37.20
[10]	74.4	74.4	85.1		
[23]	24.1	42.9	48.8		41.10
[6]	31	41.5	83		11.30
[60]	14.2	13.3	89.8		

## Data Availability

The data and materials are available with the author and available to other researchers upon request.
